# CFANet: Context Feature Fusion and Attention Mechanism Based Network for Small Target Segmentation in Medical Images

**DOI:** 10.3390/s23218739

**Published:** 2023-10-26

**Authors:** Ruifen Cao, Long Ning, Chao Zhou, Pijing Wei, Yun Ding, Dayu Tan, Chunhou Zheng

**Affiliations:** 1 Information Materials and Intelligent Sensing Laboratory of Anhui Province, School of Computer Science and Technology, Anhui University, Hefei 230601, China; rfcao@ahu.edu.cn (R.C.); ninglong1125@gmail.com (L.N.); 2 Institute of Energy, Hefei Comprehensive National Science Center, Hefei 230031, China; chao.zhou@ipp.ac.cn; 3 Institutes of Physical Science and Information Technology, Anhui University, Hefei 230601, China; weipj@ahu.edu.cn; 4 Key Laboratory of Intelligent Computing and Signal Processing of Ministry of Education, School of Artificial Intelligence, Anhui University, Hefei 230601, China; yunding@ahu.edu.cn

**Keywords:** medical image segmentation, convolution neural network, context feature fusion, attention mechanism

## Abstract

Medical image segmentation plays a crucial role in clinical diagnosis, treatment planning, and disease monitoring. The automatic segmentation method based on deep learning has developed rapidly, with segmentation results comparable to clinical experts for large objects, but the segmentation accuracy for small objects is still unsatisfactory. Current segmentation methods based on deep learning find it difficult to extract multiple scale features of medical images, leading to an insufficient detection capability for smaller objects. In this paper, we propose a context feature fusion and attention mechanism based network for small target segmentation in medical images called CFANet. CFANet is based on U-Net structure, including the encoder and the decoder, and incorporates two key modules, context feature fusion (CFF) and effective channel spatial attention (ECSA), in order to improve segmentation performance. The CFF module utilizes contextual information from different scales to enhance the representation of small targets. By fusing multi-scale features, the network captures local and global contextual cues, which are critical for accurate segmentation. The ECSA module further enhances the network’s ability to capture long-range dependencies by incorporating attention mechanisms at the spatial and channel levels, which allows the network to focus on information-rich regions while suppressing irrelevant or noisy features. Extensive experiments are conducted on four challenging medical image datasets, namely ADAM, LUNA16, Thoracic OAR, and WORD. Experimental results show that CFANet outperforms state-of-the-art methods in terms of segmentation accuracy and robustness. The proposed method achieves excellent performance in segmenting small targets in medical images, demonstrating its potential in various clinical applications.

## 1. Introduction

Medical image segmentation is a fundamental task in medical image analysis and plays an important role in clinical diagnosis, treatment planning, and disease monitoring. Traditional medical image segmentation methods involve manual annotation by experienced radiologists or physicians, which is time-consuming and prone to inter-observer variation [1]. As a result, there is a growing need for automated and accurate medical image segmentation algorithms.

In recent years, deep learning-based methods have achieved remarkable success in various medical image segmentation tasks [2,3,4,5,6,7,8]. However, the segmentation results for larger organs can reach the level of clinical experts, but the results for small targets are not high enough. In clinical medicine, there is a demand for the accurate segmentation of small targets (such as tumors, blood vessels, cells, etc.) to support medical image analysis and disease diagnosis.

**Figure 1:**
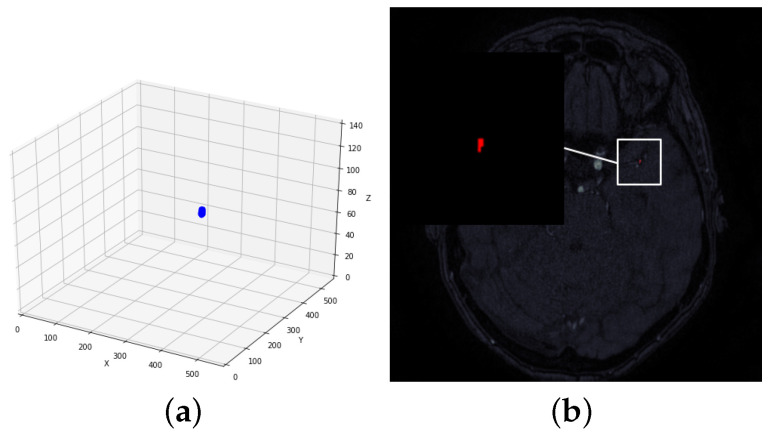
Demonstration of the difficulty of small target segmentation for intracranial aneurysms. (**a**) An example of a 3D display of a patient’s brain MRI image, highlighting an intracranial aneurysm in blue, which makes up only about 5% of all brain slices. (**b**) An example of a 2D presentation of a patient’s brain slice image, highlighting an intracranial aneurysm in red, which makes up less than 1% of all the entire image.

Taking intracranial aneurysm segmentation as an example, intracranial aneurysms are aneurysmal protrusions formed due to the disruption of the intracranial arterial blood vessel wall or local congenital defects resulting in abnormal enlargement of the local internal lumen, and ruptured intracranial aneurysms carry a high risk of death or permanent disability. In clinical practice, the segmentation of the early shape of intracranial aneurysms is very important. Figure 1 demonstrates the difficulties in segmenting intracranial aneurysms:

(1) Sparse sample: Due to the small size of intracranial aneurysms, the target region is only present in a few CT or MRI slices of each case, with the majority (90%) of slices not containing the target. As a consequence, the 2D slice dataset has a limited sample size, presenting a significant challenge for the segmentation algorithm.
(2) Small target: Intracranial aneurysms are characterized by their small size, occupying only a limited area in a single slice. Consequently, the number of pixels representing the target region is relatively small compared to the entire image. This inherent challenge lies in accurately segmenting intracranial aneurysms on a single slice. Conventional U-shaped segmentation networks often struggle to detect smaller objects effectively, and existing methods primarily focus on segmenting larger targets, lacking optimization for the detection and precise segmentation of small targets.
(3) Complex background: Small targets within an image present a notable challenge as they can be easily overshadowed by the surrounding environment, leading to potential inaccuracies in segmentation. The complexity of the background further exacerbates this issue, rendering many existing segmentation models susceptible to imprecise segmentation results.

To solve the above problems, we propose a context feature fusion and attention-mechanism-based network for small target segmentation called CFANet. Two key modules, including context feature fusion (CFF) and effective channel spatial attention (ECSA), are proposed to improve the segmentation performance based on the encoder and the decoder framework. Four datasets are adopted to validate the performance of the CFANet. The main contributions of this paper are summarized, as follows:

- We propose the Context Feature Fusion (CFF) module that merges high-dimensional features and conduct a multi-scale features extraction. High-dimensional features excel in capturing subtle variations and texture information in small targets, while the multi-scale feature extraction augments the accuracy and robustness of small target segmentation tasks. This enhances the ability to capture contextual information and depict details and shape changes of targets at different scales. Such a comprehensive target representation significantly boosts recognition and segmentation accuracy, particularly for small targets.
- We propose the Effective Channel Spatial Attention (ECSA) module, a novel approach that dynamically adjusts channel and spatial attention at the bottom of the encoder. The channel attention enhancement network improves the ability to discriminate small targets from the background, while the spatial attention focuses on the critical regions of small targets in medical images. This adaptive mechanism of ECSA enhances the characterization of small targets and mitigates the impact of background interference. The ablation experiments result show that the ECSA module could effectively improve the segmentation performance for small targets and enables the network to focus more on crucial regions for accurate segmentation.
- The performance evaluation of CFANet on four distinct small target segmentation datasets demonstrates its excellent generalization capability in accurately segmenting small targets.

## 2. Related Work

### 2.1. CNN-Based Segmentation Methods

Since the introduction of convolution neural network (CNN)-based approaches for medical image segmentation, a growing number of methods [2,3,4,5,6,7,8,9,10] have extended the standard 2D CNN architecture to solve various segmentation tasks. Ronneberger et al. [3] proposed a convolution neural network (CNN) architecture designed for medical image segmentation, called U-Net. The U-Net network structure consists of an encoding stage and a decoding stage. In the encoding process, down-sampled images are used to extract image features. In the decoding process, images are upsampled to gradually restore the original size. After up-sampling in the decoding stage, the feature maps are fused with the skip connections of the encoding information, which enriches the fine details of the image features. U-Net has become a popular and widely used model in medical image segmentation. Milletari et al. [4] proposed a V-Net segmentation framework based on the U-Net network to 3D image volume down-sampling to low resolution and used 3D convolution kernels for 3D medical images, which extends the U-Net architecture to volume segmentation by replacing 2D operations with 3D operations. Isensee et al. [8] proposed a generalized convolution neural network framework for medical image segmentation, named nnUNet, that can automatically configure the neural network structure to extract features. In addition, there are now image pyramids [11], attention mechanisms [9,10,12], separable convolution [13], large convolution kernels [14], and recurrent convolution [10] to encode the overall contextual information into a CNN-based framework. However, most recent CNN-based methods use convolution kernels for general-purpose target segmentation, while small target segmentation requires smaller convolution kernels to achieve better segmentation results.

### 2.2. Transformer-Based Segmentation Methods

The Transformer [15] is a neural network architecture primarily used in natural language processing (NLP) tasks. The key innovation of the Transformer architecture is the self-attention mechanism, which allows the network to weigh the importance of different parts of the input when making predictions. This mechanism is very effective at capturing long-range dependencies and improving model quality. Alexey Dosovitskiy et al. [16] have extended the Transformer architecture and proposed Vision-Transformer, a framework for image classification and recognition. In the context of medical image segmentation, recent works [17,18,19,20] have investigated the design of hybrid models that combine CNN with Transformer. J Chen et al. [17] introduced TransUNet, which combines the Transformer and U-Net, two classic neural network architectures, for medical image segmentation tasks. TransUNet addresses these limitations by incorporating a Transformer encoder into the U-Net architecture. The Transformer encoder enhances the model’s ability to capture global context information and long-range dependencies, resulting in an improved understanding of image semantics and structures. Another architecture, SwinUNet [19], combines the Swin Transformer and U-Net for image segmentation tasks. The Swin Transformer is a variant of the Transformer model that introduces hierarchical representations and window-based self-attention mechanisms to efficiently capture both local and global image features. Compared to TransUNet, SwinUNet greatly reduces computational complexity while achieving similar performance. Transformer-based methods achieve better segmentation performance compared to pure CNN counterparts. However, the transformer-based methods require pre-training on large-scale datasets to better exploit their advantages, while the small number of datasets targeted for small target segmentation and the large parameters are also disadvantages of the Transformer-based methods.

## 3. Methodology

### 3.1. Network Architecture

**Figure 2:**
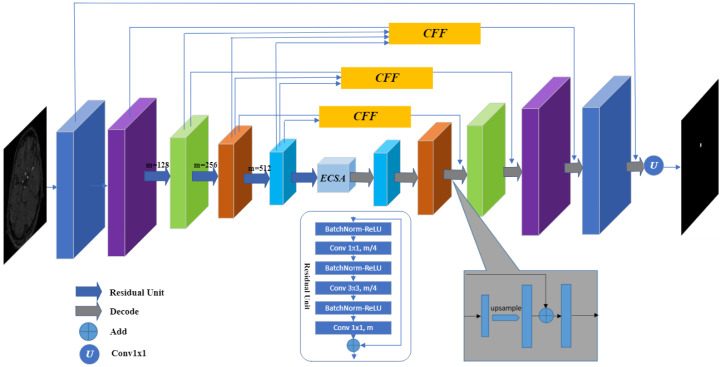
The architecture of the proposed CFANet.

Figure 2 presents the architecture of the proposed CFANet, which is based on the encoder-decoder framework and comprises four main components: encoder, context feature fusion (CFF) module, effective channel spatial attention (ECSA) module, and decoder. To enrich feature information during the encoder stage, we utilize a pre-trained ResNet34 [21] to extract image features, initializing the model with ImageNet pre-trained weights. For segmentation purposes, we exclude the average pooling layer and fully connected layers from ResNet34. The ECSA module is integrated into the lower encoder layer to enhance the model’s ability to capture precise feature information of the segmentation target. Additionally, multiple CFF modules are introduced in the skip connection section to fuse high-dimensional features extracted during the encoder stage with those from the decoder stage.

To reconstruct the original image from the feature map, we employ multiple decoder blocks in the decoder network. As illustrated in Figure 2, the decoder leverages attention features generated by the ECSA module to restore spatial information and progressively integrates high-dimensional multi-scale information from the CFF module through 3 × 3 convolutions. Each decoder block follows the subsequent steps: the input features undergo a 3 × 3 convolutional operation, and the resulting feature maps are then upsampled using bilinear interpolation. Next, the upsampled features are element-wise summed with the features from the CFF module. Upon reaching the last decoder block, the resulting feature matrix is convolved by a 1 × 1 convolutional layer to match the original input image’s dimensions.

### 3.2. Context Feature Fusion

**Figure 3:**
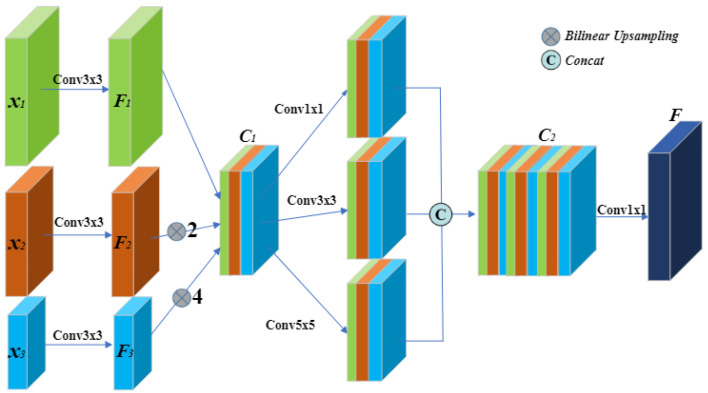
Illustration of context feature fusion (CFF) module.

On one hand, traditional U-Net’s skip connections are limited in transferring information only between neighboring layers, which may not effectively capture global context and long-distance dependencies. Although they can retain some low-level detail information, they might not be sufficient for modeling the entire image context. On the other hand, small targets, due to their small sizes and low pixel density, are relatively tiny compared to the surrounding environment. As a result, standard skip connections may not adequately capture the features of these small targets. To overcome these challenges, we introduce the Context Feature Fusion (CFF) module, as depicted in Figure 3, to enhance information transfer across different layers and improve the feature capture of small targets.

The incorporation of multi-scale feature extraction enables the network to effectively capture feature information at various scales, thereby adapting to the size variations of small targets and enhancing the detection and segmentation performance for such targets. In complex tasks, low-dimensional features may not provide sufficient information for accurate classification or prediction. In contrast, high-dimensional features contain more detailed and intricate information about the input data, making them better suited to describe complex patterns and structures. By integrating high-dimensional features, the model can retain and leverage valuable information, enhancing its representativeness and effectively handling complex tasks.

In the CFF module, instead of fusing only the features of a single stage, the skip connection will fuse the features of the current stage with all the features extracted by the encoder after the current stage. Taking the green feature matrix $x_{1}$ in Figure 2 as an example, the CFF module fuses the features of the current stage with all stages of its higher-dimensional features ($x_{2}$, $x_{3}$). The features of all stages are then passed through a 3 × 3 convolutional layer to unify the channels to match the channel size of the green feature matrix. Next, the feature matrices $F_{2}$ and $F_{3}$ are upsampled to the same size as $F_{1}$ and concatenated together. This phase of the process can be summarized as:

(1) $$C_{1}=Concat_{i=1}^{n=3}\left(f^{3\times 3}\left(x_{i}\right)⊗2^{i-n+2}\right)$$

where the $f^{n\times n}$ represents a convolution operation with the kernel size of n × n, $⊗2^{i-n+2}$ represents the up-sampling operation with rate of $2^{i-n+2}$, and Concat represents the operation of concatenation.

To capture feature information at different scales and enhance the segmentation of small targets, we draw inspiration from the SPC module in EPSANet [22], which utilizes convolution kernels of different sizes. Specifically, for $C_{1}$, we apply 1 × 1 convolutional layer, 3 × 3 convolutional layer, and 5 × 5 convolutional layer. The resulting $C_{2}$ is obtained by concatenating the feature matrices extracted at multiple scales. Finally, we use a conventional convolution to obtain the feature map of the Context Feature Fusion (CFF) module. In summary, this latter stage of the process can be summarized as:

(2) $$F=f^{1\times 1}\left(Concat_{i=1}^{k=3}\left(f^{\left(2i-1)\times (2i-1\right)}\left(C_{1}\right)\right)\right)$$

In order to reduce the effective model size and potentially speed up the inference, we keep only 3 CFF modules at the skip connections. By introducing the CFF modules, we solve the problem of large dimensional differences of segmentation targets and difficulties in multi-scale feature extraction, which can improve the segmentation accuracy and boundary clarity.

### 3.3. Effective Channel Spatial Attention

**Figure 4:**
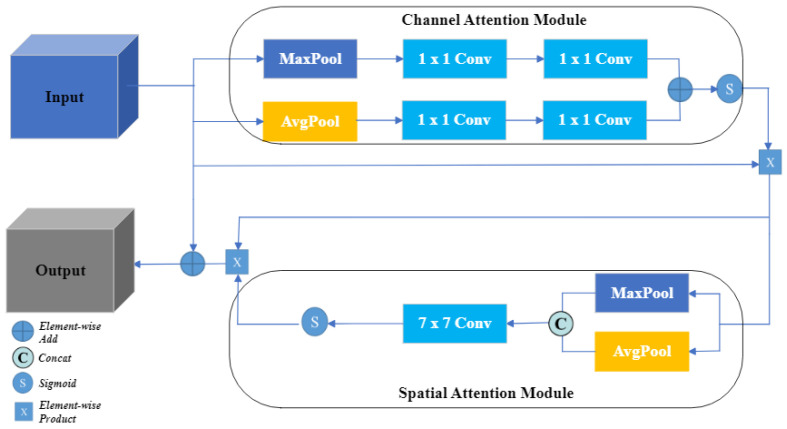
Illustration of effective channel spatial attention (ECSA) module.

As we know, small targets are easily mixed with the background in the original image, and how to make the network extract the small targets from the complex background is a pressing problem. Therefore, we add an attention mechanism called ECSA in the last part of the encoder to make the model focus on the important information in the medical image and ignore the unimportant information, as shown in Figure 4. The ECSA assigns different weights to each part of the input features and extract the critical information of the segmentation target, so that the model can make more accurate judgments without imposing more computational and storage overhead on the model.

The ECSA module mainly consists of channel and spatial attention: the channel attention mechanism adjusts the importance of each channel in the feature map by weighting each channel. The channel attention is achieved by the following steps: Firstly, we transform the input feature map *x* into scalar values for each channel by applying the maximum and average pooling operations. Next, two 1 × 1 convolutional layers are applied to the pooled feature maps to learn channel weights, and their outputs are summed up. This process compresses and then expands the channel dimension of the features, reducing complexity and enhancing generalization. Finally, the obtained weights are multiplied with the input feature map *x* and normalized using the sigmoid function to obtain the channel attention feature map $A_{channel}$. This step aims to emphasize the channel information most relevant for accurately segmenting small targets. This part of the process can be summarized by the following equation:

(3) $$A_{channel}=\sigma \left(f^{1\times 1}\left(f^{1\times 1}\left(MP\left(x\right)\right)\right)+f^{1\times 1}\left(f^{1\times 1}\left(AP\left(x\right)\right)\right)\right)$$

where the *x* represents the input feature, $f^{n\times n}$ represents a convolution operation with the kernel size of n × n, MP represents the maximum pooling operation, AP represents the average pooling operation, and σ represents the sigmoid function.

The spatial attention mechanism highlights the spatial regions where the small targets are located by weighting the different spatial locations of the feature maps, which helps the model to better capture the details and shape information of the small targets. The implementation steps for spatial attention are as follows: First, we extract the spatial information from the channel attention feature map $A_{channel}$ using maximum and average pooling, and then connect them together. Next, we learn the weights of each spatial location through a 7 × 7 convolutional layer, which are used to normalize and map the attention weights using a sigmoid function. This process results in the spatial attention feature map $A_{spatial}$, which highlights the spatial regions most relevant for the task at hand. The results of this part can be summarized by the following equation:

(4) $$A_{spatial}=\sigma \left(f^{7\times 7}\left(MP\left(A_{channel}\right)+AP\left(A_{channel}\right)\right)\right)$$

We preserve the input detail information of the original features by summing the processed feature maps with the original feature inputs in the last layer of the spatial attention module. The ECSA module equation is summarized as:

(5) $$A=x+A_{spatial}$$

We introduce the ECSA module to better focus on small targets in segmentation tasks. Channel attention enables the network to select important channel information, while spatial attention enables the network to highlight the spatial locations where small targets are located. The ECSA module enables the network to focus on small target areas and reduce the interference of the background, which can make CFANet capture and segment the features of small targets more accurately and improve the performance of segmentation.

### 3.4. Loss Function

In this section, we describe the loss function used in our proposed model. We use soft dice loss [4] as our loss function. The soft dice loss is a widely used loss function for evaluating the similarity between predicted and ground truth segmentation masks. The loss function is defined as follows:

$$L_{dice}=1-\frac{2\sum_{i=1}^{N}p_{i}\cdot t_{i}+smooth}{\sum_{i=1}^{N}p_{i}^{2}+\sum_{i=1}^{N}t_{i}^{2}+smooth}$$

where $p_{i}$ and $t_{i}$ represent the predicted and ground truth values for pixel *i*, respectively, smooth is a small constant used to smooth the denominator, and *N* is the total number of pixels.

## 4. Experiments

In this section, we evaluate the performance of the proposed method on multiple types of small target datasets and compare it with the performance of several leading networks. The datasets include: ADAM (https://adam.isi.uu.nl/, accessed on: 7 June 2022) for intracranial aneurysm segmentation from an MR image, LUNA16 (https://luna16.grand-challenge.org/, accessed on: 22 May 2023) for pulmonary nodule segmentation from CT image, Thoracic OAR (https://structseg2019.grand-challenge.org/, accessed on: 22 May 2023) for organs at a risk segmentation from CT image and WORD [23] for abdominal organ segmentation from the CT image.

We chose U-Net [3], UNet++ [24], Attention-UNet [12], ResUNet [25] and TransUNet [17] as our comparison methods. We chose these methods because they represent different network architectures and technologies. Specifically, we chose U-Net as the benchmark method because it is a commonly used and classical image segmentation network. We also chose U-Net based on the Attention Mechanism and Transformer-based network (TransUNet) because the Attention Mechanism and Transformer techniques have made significant progress in the field of medical image segmentation. Our goal is to compare the performance of these methods on small-object segmentation tasks in order to find the best segmentation method and improve the current research.

Our proposed CFANet network architecture, along with other leading network structures, are implemented on the PyTorch framework and trained using an NVIDIA RTX 3090 GPU with 24 GB of memory. To ensure optimal training, we dynamically adjust the learning rate during the training process, where the learning rate lr is calculated as lr=baselr×decline_rate. The base learning rate baselr is set to 0.01, and the decline rate decline_rate is set to 0.95. The batch size is set to 8, and we perform 150 training iterations. To optimize our model, we utilize the Adam optimizer with a weight decay of 0.00001.

We perform 5-fold cross-validation ablation experiments and comparison experiments on ADAM, LUNA16, and thoracic OAR, and ablation experiments and comparison experiments on the test set of the WORD dataset. We assess the model’s performance using two metrics: the Dice Similarity Coefficient (DSC) and the Hausdorff Distance (HD). Dice Similarity Coefficient is a statistical measure used to compare the similarity of two sets. it is defined as follows:

(6) $$DSC=\frac{2\times |A\cap B|}{\left|A|+|B\right|}$$

where *A* and *B* denote the ground truths and output probabilities, respectively.

Hausdorff Distance is a measure of similarity or distance between two sets. It represents the shortest distance between the farthest points in two sets. It is defined as follows:

(7) $$H\left(A,B\right)=\max(\underset{a\in A}{\sup}\underset{b\in B}{\inf}d\left(a,b\right),\underset{b\in B}{\sup}\underset{a\in A}{\inf}d\left(a,b\right))$$

where d(a,b) denotes the distance between pixel point *a* and pixel point *b*.

### 4.1. ADAM for Intracranial Aneurysm Segmentation

#### 4.1.1. Overview

Intracerebral aneurysms are found in 3% of the general population, and some groups have a higher risk. If an aneurysm ruptures, it may causes bleeding in the brain (subarachnoid haemorrhage) [26]. Early detection of intracranial aneurysms, as well as the accurate measurement and assessment of shape, is important in clinical routine. This enables careful monitoring of the growth and rupture risk of aneurysms to allow informed treatment decisions to be made [27]. The purpose of this Aneurysm Detection And segMentation Challenge is to automatically detect and segment intracranial aneurysms from TOF-MRA images. Automatic segmentation allows an accurate and reliable analysis of the size and shape of the aneurysms, which can help doctors to accurately identify and distinguish intracranial aneurysms from other lesions and improve the accuracy of diagnosis.

#### 4.1.2. Dataset

The ADAM dataset comprises 113 patients, including 93 with intracranial aneurysms and 20 without intracranial aneurysms. The patients in the ADAM dataset have images with varying resolutions. To demonstrate the segmentation of our proposed segmentation network for small targets, we resize the images to a resolution of 512 × 512.

#### 4.1.3. Results

**Figure 5:**
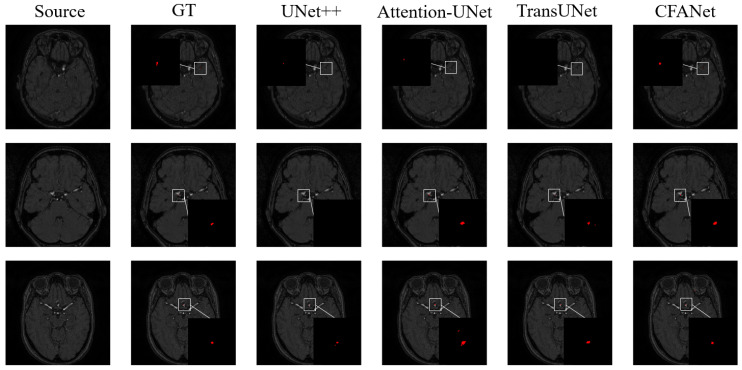
The examples of ADAM dataset segmentation. The red parts represent the segmentation results for intracranial aneurysms. From left to right: source image, ground truth (GT), UNet++, Attention-UNet, TransUNet, and our CFANet.

Table 1 shows the segmentation results on the ADAM dataset. We report the segmentation performance for small target intracranial aneurysms using DSC and HD metrics. In addition, a series of ablation experiments are performed to validate the effectiveness of the proposed CFF module and ECSA module. For better visualization, we zoom in on the small target region in the images, and Figure 5 shows our visualization results.

The highest DSC of the pure CNN-based U-Net method is 58.53%. Among the existing transformer-based methods, TransUNet [17] achieves a DSC of 55.74%. Our proposed method obtains a better performance than other existing works with a DSC of 60.74%. Moreover, in terms of HD metrics, CFANet continues to perform well. From Figure 5, we can see that CFANet segment the intracranial aneurysm completely in the figure; however, UNet++, Attention-UNet and TransUNet segment the target region blankly.

### 4.2. LUNA16 for Pulmonary Nodule Segmentation

#### 4.2.1. Overview

Lung cancer is the leading cause of cancer-related death worldwide. The National Lung Screening Trial (NLST), a randomized control trial in the U.S. including more than 50,000 high-risk subjects, showed that lung cancer screening using annual low-dose computed tomography (CT) reduces lung cancer mortality by 20% in comparison to annual screening with chest radiography [29]. In 2013, the U.S. Preventive Services Task Force (USPSTF) has given low-dose CT screening a grade B recommendation for high-risk individuals [30] and in early 2015, the U.S. Centers for Medicare and Medicaid Services (CMS) has approved CT lung cancer screening for Medicare recipients. As a result of these developments, lung cancer screening programs using low-dose CT are being implemented in the United States and other countries. Computer-aided detection (CAD) of pulmonary nodules could play an important role when screening is implemented on a large scale.

#### 4.2.2. Dataset

We apply our proposed method to the LUNA16 dataset, which excludes scans with slice thickness greater than 2.5 mm and includes a total of 888 CT scans. For the different scans, the number of scanned slices is usually small, and the size of each slice is 512 × 512 pixels. We resize the 512 × 512 image to 256 × 256 to speed up the training. After processing the images with labels, there are 2372 slices.

#### 4.2.3. Results

**Figure 6:**
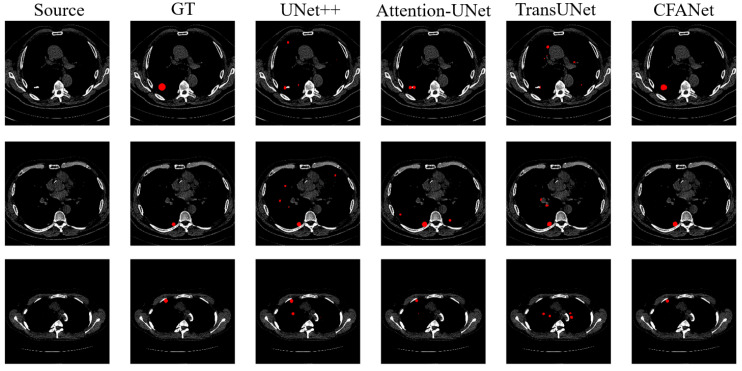
The examples of LUNA16 dataset segmentation. The red parts represent the segmentation results for pulmonary nodules. From left to right: source image, ground truth (GT), UNet++, Attention-UNet, TransUNet, and our CFANet.

Table 2 shows the segmentation results on the LUNA16 dataset. We report the segmentation performance for pulmonary nodules using DSC and HD metrics. Figure 6 shows our visualization results.

As can be observed from Table 2, CFANet achieves good results in DSC evaluation metrics compared to other methods. Although the HD metrics of TransUNet are better than ours, the HD results of the proposed CFANet are still very competitive compared with other leading methods. Figure 6 shows the segmentation results of parts of different methods on the LUNA16 dataset, and also demonstrates that the proposed CFANet is also general for small targets of lung nodules. From Figure 6, we can observe that CFANet can segment the lung nodules completely; however, most of the comparison methods segment multiple parts of the target region, which is not entirely consistent with the ground truth.

### 4.3. Thoracic OAR for Organs at Risk Segmentation

#### 4.3.1. Overview

Radiation therapy is an important cancer treatment that kills cancer cells by external irradiation. The key to treatment is to ensure that cancer cells receive enough radiation and to prevent excessive damage to normal cells in the organ at risk (OAR) [31].

The OAR may have different shapes, sizes, and locations from patient to patient, so treatment planning needs to be tailored to the patient’s characteristics. Many Small OAR such as the trachea need to be delineated accurately for designing a optimal plan, so automatic segmentation of small targets in the organ at risk is necessary.

#### 4.3.2. Dataset

We apply our proposed method to the Thoracic OAR dataset, which consists of CT images of 50 patients with segmentation targets of six organs at risk in the chest. Our primary objective is the segmentation of small targets, focusing specifically on segmenting the trachea in the dataset. The size of each slice is 512 × 512, and after processing the images with labels there are 3208 slices.

#### 4.3.3. Results

Table 3 shows the segmentation results on the Thoracic OAR dataset. We report the segmentation performance for the trachea organ using DSC and HD metrics.

**Figure 7:**
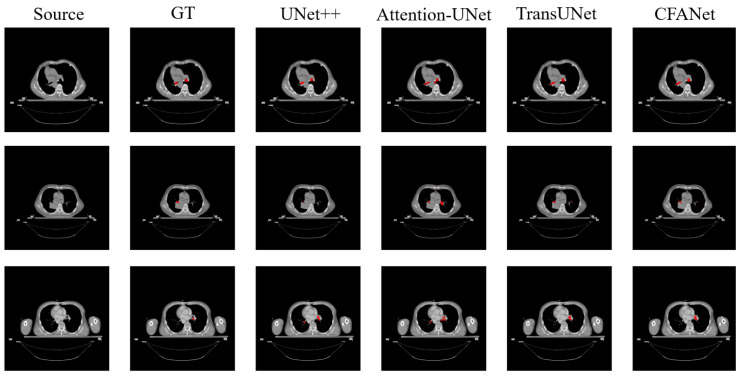
The examples of Thoracic OAR dataset segmentation. The red parts represent the segmentation results for the trachea. From left to right: source image, ground truth (GT), UNet++, Attention-UNet, TransUNet, and our CFANet.

As can be observed from Table 3, CFANet still achieves good results in DSC evaluation metrics compared to other methods. Although UNet++ is better than ours in HD metrics, our HD results are still very competitive compared to other leading methods. Figure 7 shows the segmentation results of parts of different methods on the Thoracic OAR dataset, and also demonstrates that the proposed CFANet is also general for small targets in thoracic organs. From Figure 7, we can observe that CFANet can segment the trachea of the chest organ completely, and our segmentation accuracy is higher than most of the comparison methods.

### 4.4. WORD for Abdominal Organ Segmentation

#### 4.4.1. Overview

Abdominal organ segmentation, like thoracic oar at risk segmentation, is a fundamental and important task that plays a crucial role in areas such as abdominal disease diagnosis, cancer treatment, and radiation therapy [32]. However, in clinical practice, organ segmentation is usually performed manually by radiation oncologists or radiologists. This is time-consuming and error-prone compared to the number of organs in the chest, and segmentation for small abdominal targets is particularly difficult. Therefore, segmentation of abdominal organs can be a challenging task for oncologists.

#### 4.4.2. Dataset

We apply our proposed method to the WORD dataset, a large-scale real clinical abdominal dataset (WORD), with careful annotation. All scans in the dataset are manually segmented in great detail, covering 16 organs in the abdominal region [23]. The official division of the training set, validation set, and testing set is based on a 10:2:3 ratio. To facilitate training and testing, we fuse the data from the validation set with the training set and compare the methods on the test set. In our experiments, we specifically focus on small target segmentation, and thus, we only segment the adrenal in the dataset. The number of slices in the training and test sets after processing is 2800 and 874, respectively.

#### 4.4.3. Results

Table 4 shows the segmentation results on the WORD dataset. We report the segmentation performance for abdominal organ segmentation using DSC and HD metrics.

**Figure 8:**
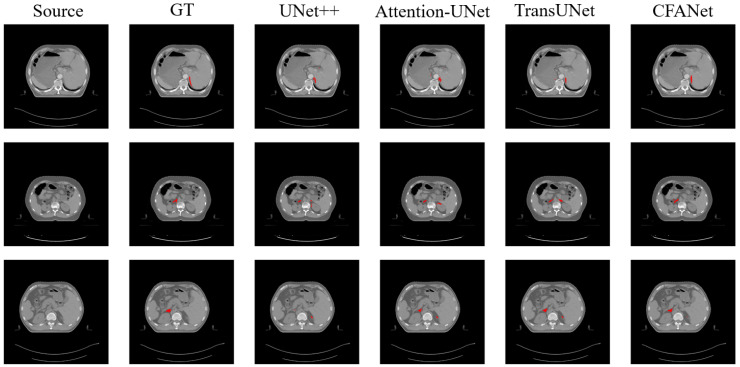
The examples of WORD dataset segmentation. The red parts represent the segmentation results for the adrenal. From left to right: source image, ground truth (GT), UNet++, Attention-UNet, TransUNet, and our CFANet.

As can be observed from Table 4, CFANet achieves the best results on DSC evaluation metrics and HD evaluation metrics compared to other methods. Figure 8 shows the segmentation results of parts of different methods on the WORD dataset, and also demonstrates that the proposed CFANet is also general for small targets in abdominal organs. From Figure 8, we can observe that CFANet can segment the adrenal of the abdominal organ completely, and our segmentation accuracy is higher than most of the comparison methods.

### 4.5. Efficiency Comparison

In this section, we conduct a comprehensive comparison of our model’s parameters and FLOPs (Floating Point Operations per Second) with respect to different architectures. Model parameters and FLOPs are critical metrics, influencing both the computational efficiency and memory requirements of a neural network.

The comparison results in Table 5 demonstrate that CFANet excels in terms of computational requirements (measured in FLOPs) compared to other models, indicating its fast inference speed. High computational demands often lead to prolonged inference times, rendering a model unsuitable for clinical applications. Although CFANet does not achieve the best result in terms of model parameters, it ranked third among most methods, showcasing a lightweight model complexity. In summary, CFANet combines higher computational efficiency with a lightweight parameter structure, making it an exceptionally appealing solution for small target segmentation in clinical medical images.

### 4.6. Ablation Study

In this experiment, we analyze the impact of the absence of key components on the network segmentation performance to verify the effectiveness of these modules. We ablate the CFF module and ECSA module in CFANet separately and apply them to these four datasets: ADAM, LUNA16, Thoracic OAR, and WORD, using DSC as an assessment metric.

We remove the proposed module and modify the architecture. From the Table 6, it can be observed that adding both modules to the proposed architecture performs better than adding only one module. In the absence of the CFF module, the poor correlation between different layers of features makes it difficult to understand the contextual information of small targets, which proves the importance of CFF module. In the absence of the ECSA module, the details and edge information of segmented targets are blurred, which proves the importance of the ECSA module. Thus, we verify that each module is effective in improving the segmentation performance of the baseline against small targets in medical images.

## 5. Conclusions

In this paper, we introduce the CFF and ECSA modules to modify the existing architecture and propose CFANet, a small target segmentation network. These modifications enable our proposed model to achieve significant performance improvements in the small target segmentation task.

By introducing the CFF module, we successfully improve information transfer and fusion. This fusion mechanism allows our model to better understand the contextual information of the small target, thus improving the accuracy and consistency of the segmentation results. The ECSA module is also introduced, and can better capture the details and edge information of the target, which is especially important for the small target segmentation task.

On the surface of the segmentation results of small targets in four medical image datasets, our proposed CFANet network outperforms the state-of-the-art networks, indicating the effectiveness and generality of our method in the field of medical image small target segmentation.

## Figures and Tables

**Table 1:** Segmentation results of different methods on ADAM.

Methods	DSC (%)	HD (mm)
U-Net [3]	58.53	10.5799
UNet++ [24]	58.29	9.7273
Attention U-Net [12]	57.18	10.0882
ResUNet [25]	53.79	8.0249
SmaAt-UNet [28]	54.62	8.5619
TransUNet [17]	55.74	11.7138
CFANet	60.74	7.7917

**Table 2:** Segmentation results of different methods on LUNA16.

Methods	DSC(%)	HD(mm)
U-Net [3]	56.29	7.3719
UNet++ [24]	56.25	7.4910
Attention U-Net [12]	64.43	7.7931
ResUNet [25]	64.08	8.0332
SmaAt-UNet [28]	62.89	7.4230
TransUNet [17]	55.78	5.2651
CFANet	65.24	6.9376

**Table 3:** Segmentation results of different methods on Thoracic OAR.

Methods	DSC(%)	HD(mm)
U-Net [3]	85.50	3.7401
UNet++ [24]	85.32	2.9489
Attention U-Net [12]	81.94	3.6448
ResUNet [25]	85.94	3.1941
SmaAt-UNet [28]	86.95	3.3427
TransUNet [17]	85.69	6.1262
CFANet	87.51	3.6094

**Table 4:** Segmentation results of different methods on WORD.

Methods	DSC(%)	HD(mm)
U-Net [3]	60.80	3.5396
UNet++ [24]	58.03	3.3330
Attention U-Net [12]	55.50	2.3379
ResUNet [25]	61.56	1.7426
SmaAt-UNet [28]	56.39	1.3403
TransUNet [17]	60.66	4.8945
CFANet	61.82	1.2252

**Table 5:** Efficiency comparison results of different methods.

Methods	Params(M)	FLOPs(G)
U-Net [3]	17	159.95
UNet++ [24]	9	137.96
Attention U-Net [12]	35	265.73
ResUNet [25]	52	82.99
SmaAt-UNet [28]	4	78.05
TransUNet [17]	66	85.64
CFANet	30	59.24

**Table 6:** Segmentation results of ablation experiments.

Dataset	Methods	DSC(%)
ADAM	Baseline	58.00
	Baseline + CFF	59.26
	Baseline + ECSA	58.56
	CFANet	60.74
LUNA16	Baseline	64.17
	Baseline + CFF	64.25
	Baseline + ECSA	64.51
	CFANet	65.24
Thoracic OAR	Baseline	86.24
	Baseline + CFF	86.37
	Baseline + ECSA	87.26
	CFANet	87.51
WORD	Baseline	59.28
	Baseline + CFF	59.65
	Baseline + ECSA	59.70
	CFANet	61.82

## Data Availability

Not applicable.
